# II – Avaliação do Controle Autonômico da Frequência Cardíaca em Diversas Condições Clínicas

**DOI:** 10.36660/abc.20250112

**Published:** 2026-01-09

**Authors:** Benedito Carlos Maciel, Lourenço Gallo-Jr, André Schmidt, José Antonio Marin-Neto

**Affiliations:** 1 Universidade de São Paulo Ribeirão Preto SP Brasil Universidade de São Paulo – Campus Ribeirão Preto, Ribeirão Preto, SP – Brasil

**Keywords:** Doenças do Sistema Nervoso Autônomo, Frequência Cardíaca, Sistema Nervoso Simpático, Sistema Nervoso Parassimpático

## Abstract

Por meio da comparação das respostas de voluntários normais, a avaliação da função autonômica em condições clínicas anormais se focou na detecção de disautonomia envolvendo os sistemas parassimpático e adrenérgico em pacientes com cardiopatia chagásica, pós-cirurgia cardíaca, insuficiência cardíaca crônica, prolapso da valva mitral e hipertireoidismo. Em particular, o comprometimento autonômico observado em pacientes com doença de Chagas envolveu predominantemente o controle parassimpático da frequência cardíaca ao nível do nó sinusal e a inervação adrenérgica ao nível ventricular do miocárdio. Os distúrbios autonômicos observados em pacientes com cardiomiopatia chagásica só recentemente foram explorados em termos de sua relevância prognóstica, e suas potenciais implicações clínicas para fins terapêuticos ainda precisam ser investigadas.

Ao longo das últimas quase sete décadas, nosso laboratório acumulou expertise significativa utilizando diversos testes descritos acima para avaliar o controle autonômico da frequência cardíaca, agora com foco em diversas condições clínicas fisiopatológicas. O efeito do treinamento físico de resistência e do envelhecimento foi focado principalmente em estudos com voluntários normais, cujas respostas basais serviram como controles a serem comparados durante testes empregados em indivíduos com algumas condições clínicas mórbidas. De longe, a fisiopatologia da cardiopatia chagásica envolvendo o sistema nervoso autônomo foi o assunto mais predominantemente estudado em nosso laboratório, desde os primeiros estudos na década de 1960 até os estudos tardios recentemente publicados em periódicos internacionais (Figura Central). Outras condições patológicas focadas em nossos estudos foram prolapso da valva mitral, insuficiência cardíaca, pós-cirurgia cardíaca e hipertireoidismo.

## Graphical abstract

**Figura Central:**
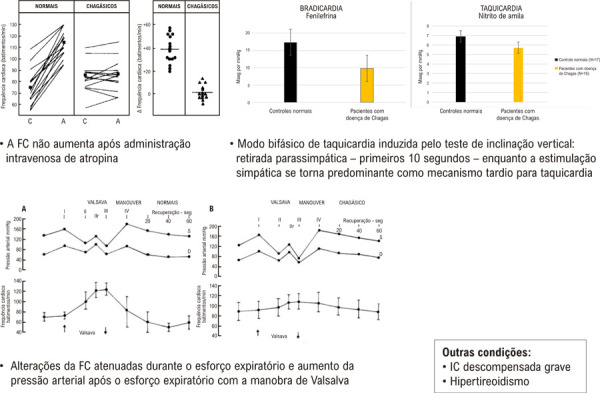
II – Avaliação do Controle Autonômico da Frequência Cardíaca em Diversas Condições Clínicas

Avaliação do controle autonômico da frequência cardíaca na disautonomia da doença de Chagas.

## Disautonomia na doença de Chagas

**Figura 1:**
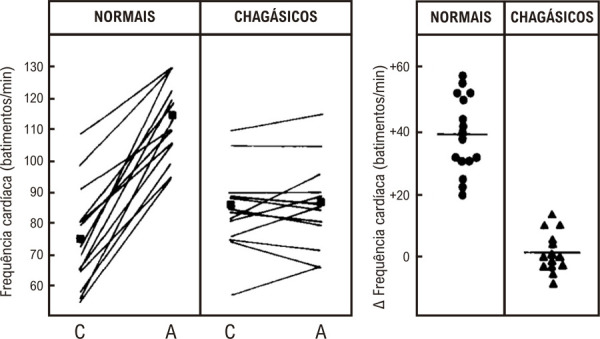
– Efeitos do sulfato de atropina na frequência cardíaca (administração venosa periférica na dose de 0,04 mg/kg). Valores antes (C) e após o bloqueio vagal (A). Valores absolutos e médios (painel esquerdo) e alterações incrementais individuais e médias (painel direito).12

De acordo com a denervação parassimpática anatômica descrita por vários pesquisadores independentes relatando estudos morfológicos, a regulação autonômica cardíaca anormal foi demonstrada conclusivamente em pacientes com doença de Chagas (DC).^1^Vários estímulos farmacológicos e fisiológicos foram utilizados para demonstrar uma regulação da frequência cardíaca parassimpática notavelmente prejudicada. A injeção intravenosa de fármacos anti-hipertensivos, como metaraminol e fenilefrina, demonstrou, em pacientes com DC, produzir um aumento transitório comparável da pressão arterial sistêmica, sem um grau semelhante de bradicardia reflexa, em comparação com indivíduos controle normais. Pacientes com DC também não responderam com aumento da frequência cardíaca (FC) à administração intravenosa de atropina. A disautonomia evidenciada com esses testes farmacológicos foi corroborada por outros testes fisiológicos, incluindo imersão facial em água, manobra de Valsalva, testes de inclinação da cabeça para cima e para baixo, arritmia sinusal respiratória (ASR), preensão manual, exercício dinâmico (ED) gradual e análise espectral de registros de Holter.^2 - 8^ No geral, esses estudos mostraram que a maioria dos pacientes com DC geralmente são privados da ação inibitória tônica normalmente exercida pelo sistema parassimpático no nó sinusal.^9 , 10^( Figura 1 ). Além disso, esses pacientes com DC não possuem o mecanismo mediado vagalmente para responder com bradicardia ou taquicardia reflexa rápida por meio da retirada parassimpática a alterações transitórias na pressão arterial ou no retorno venoso. Além disso, esses estudos abriram caminho para o conceito da DC como modelo experimental espontâneo adequado para estudar uma cardiomiopatia com uma disfunção autonômica específica no nível do nó sinusal.^10^

É importante enfatizar que a disautonomia na DC é um achado precoce na história natural da doença, visto que, por exemplo, respostas anormais da FC ao bloqueio parassimpático intravenoso com atropina têm sido consistentemente demonstradas na DC, precedendo quaisquer sinais de disfunção sistólica ventricular ou insuficiência cardíaca. Em contraste, o bloqueio simpático farmacológico com propranolol intravenoso produziu graus semelhantes de elevação da FC em pacientes com DC e controles normais.^9 , 10^

**Figura 2:**
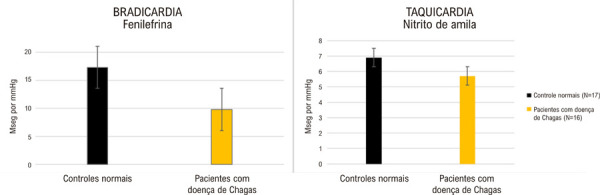
– Sensibilidade dos barorreceptores durante alterações agudas induzidas na resistência vascular sistêmica por fenilefrina e nitrito de amila. Observe as diferenças marcantes entre pacientes normais e portadores de doença de Chagas cardíaca durante a hipertensão induzida por fenilefrina; as diferenças durante a hipotensão com nitrito de amila foram menos significativas.10

A sensibilidade do barorreflexo foi avaliada usando o método descrito por Pickering et al.,^11^relacionando os intervalos de pulso batimento a batimento com os valores de pressão sistólica imediatamente precedentes durante alterações transitórias na pressão arterial sistêmica induzidas por fenilefrina ou nitrito de amila. Em comparação com controles normais, tanto a bradicardia quanto a taquicardia observadas em pacientes com DC foram significativamente atenuadas. No entanto, a bradicardia reflexa rápida induzida pela hipertensão induzida por fenilefrina foi claramente mais deprimida do que a hipotensão da taquicardia reflexa rápida causada pelo nitrito de amila. Esses achados foram interpretados como indicativos de que o comprometimento autonômico em pacientes com DC afetou predominantemente o sistema parassimpático – responsável pela bradicardia, enquanto o sistema simpático – responsável pela taquicardia, que ocorre mais tarde, pôde ser relativamente poupado^10^ ( Figura 2 ).

**Figura 3:**
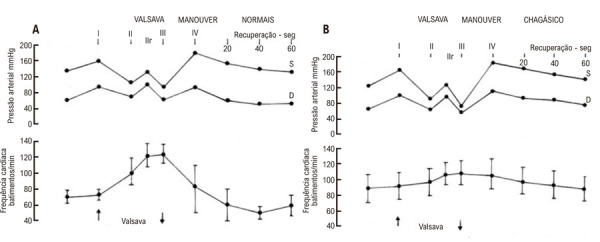
– Valores de pressão arterial (média) e frequência cardíaca (média e desvio-padrão) durante a manobra de Valsalva (40 mm Hg por 20 segundos) em grupos de indivíduos normais (A; n = 18) e pacientes com cardiopatia chagásica (B; n = 14). Em comparação com controles normais, os pacientes com doença de Chagas apresentam alterações discretas na frequência cardíaca durante o esforço e aumento da pressão arterial após a manobra de Valsalva. No grupo de pacientes chagásicos, não há bradicardia absoluta e há também um retorno mais tardio da pressão arterial aos valores basais após o esforço. Os pacientes chagásicos incluídos neste estudo foram selecionados devido à ausência de resposta cronotrópica à atropina (0,04 mg/kg–1 intravenosamente).1

Essas alterações cronotrópicas atenuadas, utilizando testes farmacológicos, foram corroboradas por aquelas observadas em resposta à manobra de Valsalva (40 mmHg por 20 segundos) em pacientes com DC que não apresentaram taquicardia em resposta à infusão intravenosa de atropina. Apesar de apresentarem valores de sobretensão comparáveis após a liberação da manobra de Valsalva, os pacientes com DC que não apresentaram aumento da FC após a atropina também não apresentaram bradicardia absoluta, e houve também um retorno mais tardio da pressão arterial aos valores basais após a manobra de Valsalva^1^( Figura 3 ).

**Figura 4:**
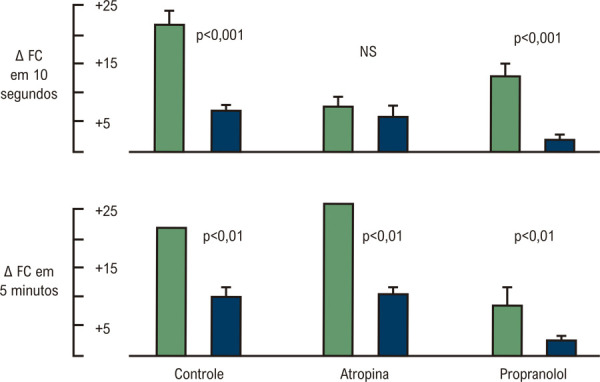
– Efeitos da inclinação de indivíduos normais (barras verdes) e pacientes com doença de Chagas (barras azuis) para a posição de cabeça erguida, antes e depois da administração de atropina e propranolol. Em cada caso, o valor médio é indicado pela barra horizontal e o erro padrão da média pela linha horizontal na parte superior da barra. Delta FC = incremento da frequência cardíaca, 10 s e 5 min após as mudanças posturais.12

A taquicardia reflexa induzida pela mudança da posição supina para uma inclinação de 70° da cabeça para cima foi estudada em indivíduos normais conscientes e pacientes com cardiopatia chagásica crônica na ausência de insuficiência cardíaca. Pacientes com DC apresentaram respostas de FC marcadamente diminuídas durante os 10 segundos iniciais após a inclinação para a postura ereta. Uma resposta semelhante foi obtida em indivíduos normais após bloqueio parassimpático com atropina. O bloqueio beta-adrenérgico não produziu um efeito significativo na resposta inicial da FC de indivíduos normais, mas o incremento da frequência cardíaca, em 1 e 5 minutos de inclinação, foi significativamente reduzido em indivíduos normais e abolido em pacientes. Esses resultados em pacientes com DC corroboram o que havia sido encontrado em indivíduos normais, de um modo bifásico de taquicardia provocado pela postura ereta: inicialmente, depende da retirada parassimpática – um mecanismo mais deprimido nos pacientes – enquanto a estimulação simpática se torna o mecanismo tardio predominante para taquicardia na posição ortostática^12^ ( Figura 4 ). Nestes estudos também foi observado que, embora menos acentuado, os pacientes com DC também apresentam alguma depressão da resposta adrenérgica à inclinação vertical.

**Figura 5:**
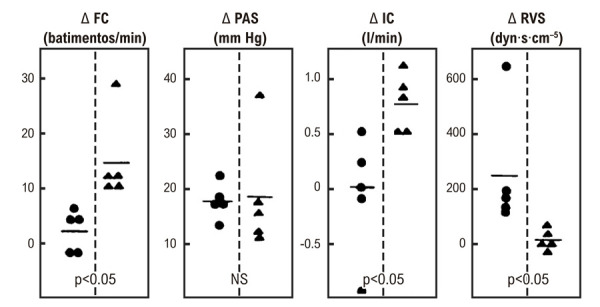
– Alterações na frequência cardíaca (FC), pressão arterial sistêmica média (PAS), índice cardíaco (IC) e resistência vascular sistêmica (RVS) evocadas pela preensão manual a 30% da contração voluntária máxima em pacientes com doença de Chagas com (círculos) e sem comprometimento parassimpático (triângulos). A significância estatística foi avaliada pelo teste-t de Student para amostras não pareadas.13

No entanto, nem todos os pacientes com DC apresentam o controle parassimpático anormal da FC descrito nos estudos acima. Que esse conceito é verdadeiro foi demonstrado em outras investigações do nosso laboratório, quando as respostas hemodinâmicas ao exercício isométrico sustentado (preensão manual a 30% da capacidade voluntária máxima) foram estudadas em diferentes grupos de pacientes com cardiopatia chagásica, todos sem insuficiência cardíaca prévia ou atual. Um grupo (1) de pacientes apresentou comprometimento profundo do controle parassimpático da frequência cardíaca, considerando que não apresentaram taquicardia em resposta à administração intravenosa de atropina e nem bradicardia durante a fase IV da manobra de Valsalva. O outro grupo (2) apresentou regulação vagal normal da frequência cardíaca, avaliada pelas respostas cronotrópicas a esses testes. A alteração da FC induzida pelo teste de preensão manual foi significativamente menor no grupo 1 do que no grupo 2. As respostas pressoras e o índice sistólico à preensão manual foram de magnitude semelhante em ambos os grupos. O débito cardíaco indexado para a área de superfície corporal aumentou durante a preensão manual no grupo 2, mas não houve alteração significativa no grupo 1. As alterações na resistência vascular sistêmica medida foram significativamente maiores no grupo 1 do que no grupo 2. Este estudo também demonstrou que o comprometimento parassimpático está associado a efeitos adversos mais evidentes quando há depressão inotrópica do miocárdio, como demonstrado pela sobrecarga hemodinâmica causada por exercício isométrico em pacientes com cardiopatia chagásica. Nessas condições, a resposta da pressão arterial à preensão manual é predominantemente mediada por um aumento na resistência vascular sistêmica, em vez de um aumento no débito cardíaco^13^( Figura 5 ).

**Figura 6:**
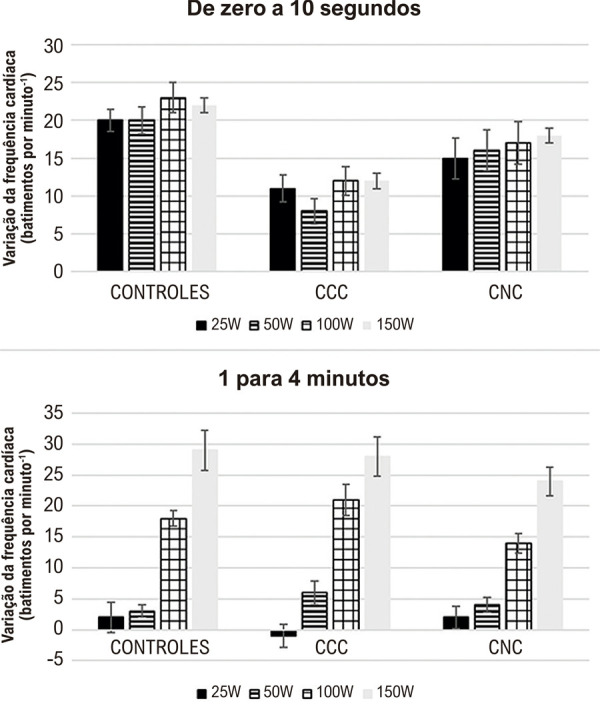
– Alterações na frequência cardíaca induzidas por exercício dinâmico em a) 0-10 s e b) 1-4 min em todas as cargas de trabalho (25, 50, 100 e 150 W) em indivíduos normais e em pacientes com doença de Chagas cardíaca e não cardíaca. As linhas contínuas indicam as medianas, as linhas tracejadas o primeiro e o terceiro quartis e as linhas verticais os dados mais extremos.14 CCC: Doença de Chagas cardíaca; CNC: Doença de Chagas não cardíaca.

O ED foi utilizado com um cicloergômetro eletromagnético com frenagem, para avaliar as respostas da FC em relação às outras variáveis cardiorrespiratórias (ventilação pulmonar (V), consumo de oxigênio (VO2), produção de dióxido de carbono (VCO2) e quociente respiratório (QR)) comparando indivíduos normais com dois grupos de pacientes com DC crônica (com e sem cardiomiopatia, ou seja, com a forma indeterminada da doença). A evolução temporal das respostas da FC ao aumento da carga de trabalho foi utilizada para avaliar as condições funcionais dos sistemas eferentes cardíacos simpáticos e parassimpáticos. As análises dos resultados mostraram que (a) o grupo de pacientes com DC e cardiomiopatia evidente (eletrocardiograma anormal, mesmo com tamanho cardíaco normal nas radiografias de tórax e sem insuficiência cardíaca prévia) apresentou incrementos menores na FC (p < 0,05) do que indivíduos normais durante os 10 s iniciais de cada carga de exercício (delta FC 0-10 s) de esforço (componente rápido do aumento da FC); (b) as diferenças entre os indivíduos de controle normais e os pacientes com DC sem cardiomiopatia não foram estatisticamente significativas; (c) as anormalidades nas respostas cronotrópicas são consistentes com o comprometimento da modulação eferente parassimpática no nó sinusal; (d) em contraste, a resposta lenta da FC (delta FC 1-4 min), que expressa o grau de estimulação simpática no nó sinusal, foi comparável nos três grupos estudados, mostrando assim respostas adrenérgicas não prejudicadas durante o ED no grupo de pacientes com a forma indeterminada de DC; e (e) os valores de VO2, VCO2 e QR foram normais em todas as cargas de trabalho em ambos os grupos de indivíduos com DC, sugerindo que a disfunção vagal não afeta o transporte de oxigênio nesses níveis submáximos de ED^14^( Figura 6 ).

**Figura 7:**
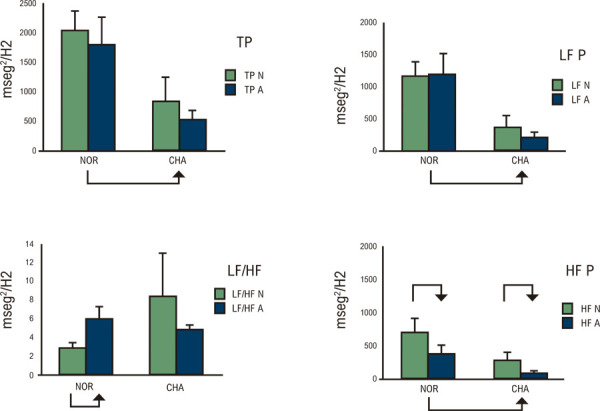
– Variabilidade espectral de 24 horas da frequência cardíaca registrada em indivíduos controle (NR) e em pacientes com doença de Chagas crônica (NA). Média e desvio-padrão para os dois grupos. As barras verdes e em blocos indicam os valores de sono (noite) e vigília (diurno), respectivamente. No sentido horário, a partir do canto superior esquerdo: TP (potência total), LFP (potência de baixa frequência), HFP (potência de alta frequência) e LF/HF (razão baixa/alta frequência). Pacientes com doença de Chagas apresentaram valores inferiores aos normais, além da ausência da relação LF/HF esperada ao despertar.28

Também utilizamos a análise espectral de potência da variabilidade da frequência cardíaca (VFC), uma técnica não invasiva extremamente confiável para avaliar a influência autonômica sobre o nó sinusal em pacientes com DC ( Figura 7 ). Uma redução significativa na potência da variabilidade cronotrópica noturna e matinal, afetando tanto os componentes de baixa quanto de alta frequência, foi demonstrada pela análise espectral de registros eletrocardiográficos de Holter de 24 horas em pacientes com DC. Assim, a atenuação de ambos os componentes durante a análise espectral da variabilidade cronotrópica em pacientes com DC é consistente com a ocorrência de disfunção simpática e parassimpática. Além disso, essa VFC anormal foi documentada em pacientes com DC em repouso, em pé ou realizando exercícios de preensão manual.^8^

Como já foi apontado acima, a disfunção autonômica em pacientes com DC pode ser detectada antes do desenvolvimento da disfunção ventricular e em todas as fases da doença, mesmo naqueles indivíduos com as formas digestivas indeterminadas e isoladas.^7 , 15 , 16^ No entanto, em alguns dos nossos estudos, houve uma tendência clara de pacientes apresentando disfunção cardiovascular parassimpática apresentarem o envolvimento típico do órgão digestivo da DC, ou seja, megaesôfago e/ou megacólon, cuja fisiopatologia é considerada principalmente devido à denervação intramural parassimpática visceral.^17 - 21^

**Figura 8:**
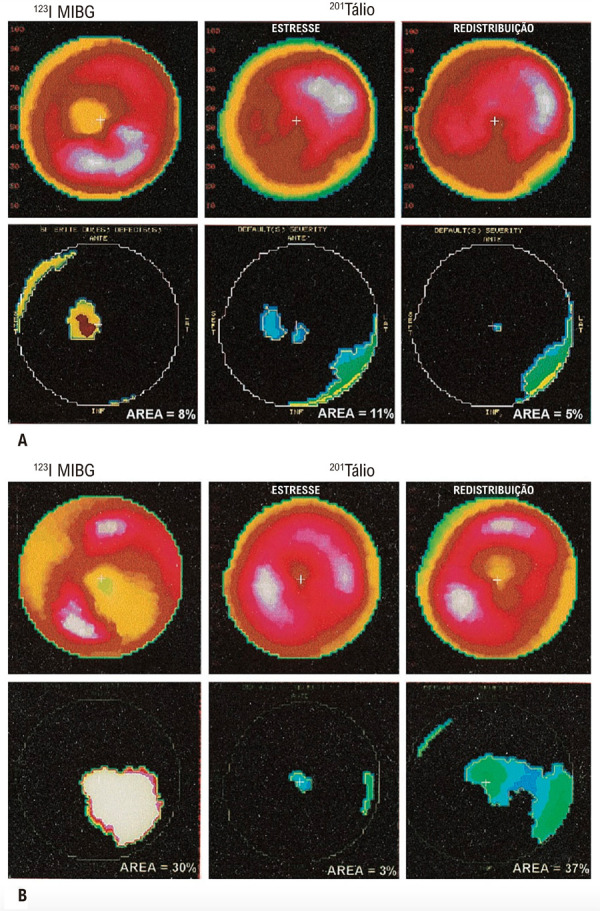
– Exemplos de estudos de cintilografia miocárdica utilizando 123I-MIBG (coluna da esquerda) e estresse e/ou redistribuição de tálio-201 (colunas do centro e da direita, respectivamente). Representação em mapa polar da porcentagem de captação miocárdica (relacionada à densidade máxima de contagem de pixels) (linha superior) e o resultado da análise quantitativa mostrando áreas de captação reduzida (> 2,5 DPs em comparação aos valores médios do grupo controle) (linha inferior). (A) Homem de 37 anos, paciente com doença de Chagas do grupo I (fração de ejeção do VE de 50%). A captação reduzida de 123I-MIBG nas áreas septais anteroseptal e apical está topograficamente relacionada ao distúrbio reversível da perfusão miocárdica. (B) Mulher de 54 anos, paciente com doença de Chagas do grupo II (fração de ejeção do VE de 52%). Grande área de captação reduzida de 123I-MIBG nas paredes inferior, posterior e apical, topograficamente relacionada a defeito de perfusão fixo e/ou paradoxal moderado no estudo com tálio-201. Também houve comprometimento grave da motilidade da parede nos segmentos envolvidos.23

Embora a teoria neurogênica tenha sido proposta como o mecanismo patogênico fundamental para a cardiomiopatia chagásica,^22^ a patogênese da cardiomiopatia chagásica é definitivamente mais complexa, e a teoria neurogênica para a cardiomiopatia chagásica encontrou vários obstáculos conceituais insolúveis. Além disso, a detecção de disautonomia cardíaca em todos esses estudos se baseou essencialmente na avaliação de respostas de FC prejudicadas, claramente causadas por danos no suprimento neuronal às estruturas sinoatriais. Portanto, distúrbios adrenérgicos que ocorrem no nível miocárdico ventricular podem ter sido negligenciados. Um estudo mais detalhado de nosso laboratório se concentrou na cintilografia com metaiodobenzilguanidina marcada com iodo-123 (^123^I-MIBG) para avaliação da inervação nervosa simpática miocárdica e mostrou denervação simpática segmentar que foi detectada mesmo em pacientes com a forma indeterminada da cardiopatia chagásica, precedendo anormalidades no movimento da parede do ventrículo esquerdo (VE)^23^( Figura 8 ). Um achado intrigante adicional desse estudo foi o aumento da taxa de eliminação de ^123^I-MIBG observada em pacientes com função ventricular normal.^24^ Isso pode ser resultado do aumento paradoxal da atividade simpática cardíaca nos estágios iniciais da DC.

É digno de nota que a disautonomia circulatória encontrada em muitos pacientes com DC difere de outras síndromes que afetam o sistema nervoso autônomo (por exemplo, diabetes mellitus, amiloidose nervosa de Andrade), uma vez que nenhuma anormalidade significativa do controle vascular adrenérgico que leve a sintomas ou evidência objetiva de hipotensão postural foi relatada nesses estudos. Além disso, não houve demonstração clara de que o comprometimento autonômico per se seja prognosticamente relevante, e nenhuma implicação terapêutica foi concebida. De fato, embora o comprometimento autonômico tenha sido há muito tempo hipotetizado como um mecanismo potencial que causa morte súbita em pacientes com DC,^1^ uma ligação entre a variabilidade reduzida da FC e um prognóstico mais preocupante associado a um escore de Rassi alto,^25^ foi relatado apenas recentemente. Além disso, investigações recentes do nosso laboratório forneceram evidências de que a extensão da denervação adrenérgica miocárdica irregular detectada com ^123^I-MIBG está correlacionada com a ocorrência e a gravidade de arritmia ventricular maligna em pacientes com cardiomiopatia chagásica crônica.^26^

Vale ressaltar que o grau de comprometimento autonômico é bastante variável de indivíduo para indivíduo no contexto da DC e, em muitos estudos, sua magnitude foi muito sutil; portanto, diferenças minúsculas em comparação aos controles normais podem ter sido superinterpretadas. Comparando a disfunção autonômica do nó sinusal detectada por muitos testes diferentes, com a disfunção simpática do VE avaliada pela cintilografia com ^123^I-MIBG, em diferentes grupos de DC estudados em nosso laboratório, parece que cada um é de natureza independente e não interrelacionado. No geral, investigações independentes apontam para quatro mecanismos patogênicos principais para explicar o desenvolvimento da cardiomiopatia chagásica crônica, um dos quais é, sem dúvida, a disautonomia, e o outro inclui distúrbios microvasculares, agressão miocárdica dependente de parasitas e lesão miocárdica imunomediada. Apesar de suas peculiaridades proeminentes, o papel dos distúrbios autonômicos e dos distúrbios microcirculatórios é provavelmente auxiliar entre as principais causas de dano miocárdico crônico. A patogênese da cardiopatia chagásica crônica é, em essência, dependente de uma infecção sistêmica de baixo grau, mas incessante, com reação imunológica adversa documentada. Portanto, a persistência parasitária e os mecanismos imunológicos estão inextricavelmente e causalmente relacionados à agressão miocárdica observada na fase crônica da cardiopatia chagásica.^1^

Em essência, muitas investigações que empregam diversos testes que avaliam o controle autonômico da frequência cardíaca, com métodos farmacológicos e fisiológicos, demonstraram distúrbios acentuados do sistema parassimpático e, em menor grau, também do sistema adrenérgico em pacientes com todas as formas de DC crônica. Além disso, em nível ventricular, distúrbios no sistema adrenérgico puderam ser detectados e, mais relevante, essas alterações podem ter possível papel no desencadeamento ou agravamento de arritmias cardíacas.

## Regulação autonômica da frequência cardíaca na síndrome de insuficiência cardíaca crônica devido a várias etiologias

Desde a década de 1970, duas investigações independentes relataram controle parassimpático e simpático autonomicamente mediado anormal da FC em pacientes com disfunção ventricular ou insuficiência cardíaca congestiva (ICC).^27 , 28^ No entanto, em contraste com o que foi encontrado em nossos estudos com pacientes cuja disautonomia era devida à DC e ocorria na ausência de disfunção contrátil cardíaca, essas investigações sobre ICC não puderam relatar nenhuma evidência patológica de anormalidades na inervação anatômica cardíaca. Portanto, o comprometimento do controle barorreflexo da FC descrito em pacientes com ICC de qualquer etiologia poderia ser funcionalmente determinado, ser inespecífico e também possivelmente reversível. Para determinar se esse distúrbio nesse cenário clínico de ICC poderia ser reversível, nosso laboratório se propôs a avaliar o controle cronotrópico cardíaco em pacientes com ICC crônica classe IV de várias etiologias antes e após a compensação clínica obtida por repouso no leito, restrição de sal, diuréticos e vasodilatadores. O manejo foi modificado 3 dias antes da segunda avaliação autonômica, de modo a restabelecer a mesma dieta e condições farmacológicas do estudo basal anterior. A compensação clínica documentada levou a uma redução significativa na classe NYHA baseada em sintomas, peso corporal e congestão pulmonar e sistêmica. As respostas médias às alterações da FC (batimentos/min) aumentaram significativamente após a compensação em todos os pacientes testados com atropina, preensão manual (capacidade máxima de 30%) e inclinação da cabeça para cima (5 minutos). Uma inclinação barorreflexa acentuadamente atenuada foi confirmada no estado descompensado da ICC, de acordo com os testes com fenilefrina e nitrito de amila. Um aumento notável na sensibilidade barorreflexa ocorreu após a compensação, tanto nos testes com fenilefrina quanto com nitrito de amila. Esses achados documentaram, pela primeira vez na literatura, um componente reversível do controle barorreflexo prejudicado da FC na ICC grave de etiologia inespecífica, possivelmente devido aos seus pronunciados efeitos congestivos.^29^

Em resumo, esta investigação demonstrou que após a compensação clínica da insuficiência cardíaca, houve uma melhora significativa no controle autonômico da FC documentada por testes com atropina, preensão manual e inclinação da cabeça, bem como por um aumento notável na sensibilidade do barorreflexo.

## Controle autonômico da frequência cardíaca em pacientes tratados com cirurgia cardíaca sob circulação extracorpórea

**Figura 9:**
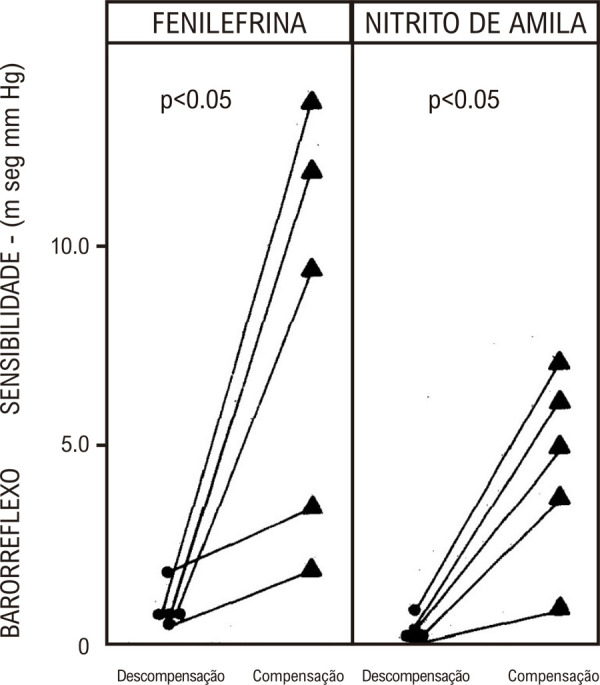
– Valores individuais de sensibilidade do barorreflexo obtidos com fenilefrina e nitrito de amila durante a descompensação (DECOMP.) e compensação (COMP.) da insuficiência cardíaca congestiva.29

A sensibilidade do reflexo barorreflexo à hipertensão transitória foi determinada em pacientes antes (controle) e depois (72 horas) de operações cardíacas abertas com circulação extracorpórea (CEC). Em todos os pacientes, a avaliação pós-operatória precoce da sensibilidade do barorreflexo mostrou valores que foram apreciavelmente diminuídos (p < 0,01) em comparação com os valores de sensibilidade pré-operatória ( Figura 9 ). A sensibilidade embotada sugeriu comprometimento grave do controle barorreflexo do nó sinoatrial na condição pós-operatória. O controle barorreflexo prejudicado não se correlacionou com alterações concomitantes na FC ou nas pressões arteriais sistêmicas, atriais esquerda ou direita. Além disso, a arritmia do nó sinusal respiratório estava ausente em todos os indivíduos. Em quatro pacientes, estudos subsequentes em 4, 8, 10 e 12 meses, respectivamente, após a operação de coração aberto, revelaram boa recuperação da sensibilidade do barorreflexo e influências respiratórias na variação batimento a batimento. Esses achados apontam para um comprometimento ainda maior do controle fino da FC imposto pelas condições das operações cardíacas com CEC em pacientes submetidos a essa modalidade de cirurgia torácica.^30^

Resumindo, este estudo mostrou que 72 horas após cirurgia cardíaca com CEC, os pacientes apresentaram comprometimento acentuado do controle barorreflexo do nó sinoatrial e abolição da ASR, mas apresentaram boa recuperação desses controles reflexos da FC entre 4 e 12 meses após a cirurgia.

## Regulação autonômica da frequência cardíaca em pacientes com hipertireoidismo

Os mecanismos que controlam a FC em repouso no hipertireoidismo foram avaliados em pacientes antes e depois do tratamento com propiltiouracil. Os pacientes em repouso foram estudados em condições basais e após bloqueio farmacológico autonômico em duas sessões experimentais: primeira sessão, usando propranolol (0,2 mg/kg de peso corporal); segunda sessão, usando atropina (0,04 mg/kg de peso corporal) seguida de propranolol (0,2 mg/kg de peso corporal). Todos os fármacos bloqueadores farmacológicos foram administrados por via intravenosa. A FC em repouso no início do estudo foi significativamente reduzida após o controle clínico e laboratorial da doença. Após o bloqueio duplo, a FC intrínseca foi reduzida após o tratamento (p < 0,025). A redução da FC causada pelo propranolol não foi significativamente diferente antes e depois do controle clínico do hipertireoidismo com propiltiouracil. Em contraste, a atropina induziu uma elevação maior da FC após o tratamento do que antes do tratamento clínico. Os presentes resultados não sugerem participação apreciável do componente simpático do sistema nervoso autônomo na taquicardia do hipertireoidismo, pelo menos nas condições do presente estudo. A pequena alteração observada na FC intrínseca, embora significativa, parece indicar que este não é o mecanismo mais importante envolvido na taquicardia exibida por pacientes com hipertireoidismo. Além disso, nossos resultados sugerem que uma redução importante na atividade eferente do componente parassimpático participa dos mecanismos que modulam a FC em repouso no hipertireoidismo.^31^ Esta conclusão foi confirmada quando avaliamos a magnitude da ASR em pacientes com hipertireoidismo, antes e após o tratamento, mostrando que as alterações cardíacas induzidas por esta manobra fisiológica aumentaram significativamente em todos os pacientes, indicando um comprometimento importante e reversível da atividade vagal eferente sobre o nó sinusal no hipertireoidismo humano.^32^

Também investigamos a contribuição relativa dos componentes simpáticos e parassimpáticos do sistema nervoso autônomo para a regulação da resposta cronotrópica ao ED na posição sentada (por 4 min em cicloergômetro com freio eletromagnético nos níveis de 5, 15, 25, 50, 75 W) que foi avaliado indiretamente em pacientes com tireotoxicose, antes e após o controle clínico da doença, e comparado a voluntários normais, bem como sob bloqueio farmacológico simpático com propranolol (0,2 mg/kg de peso corporal).^33^

A magnitude do aumento líquido na FC evocada por cada nível de ED foi equivalente em pacientes normais e hipertireoidianos, mas nesses pacientes ocorreu um incremento atenuado no início do exercício, que depende de um mecanismo de retirada predominantemente vagal; em contraste, para cada carga de trabalho, após 30 s de esforço, quando a contribuição simpática se torna o mecanismo predominante para elevação da FC, o incremento da FC foi maior em pacientes hipertireoidianos em comparação a indivíduos normais.

Além disso, documentamos que, em pacientes com hipertireoidismo, o bloqueio β-adrenérgico deprimiu a taquicardia após 30s de esforço nos níveis de 15 e 50 W, enquanto em indivíduos normais esse efeito se manifestou apenas em 50 e 75 W. Além disso, após a compensação clínica da doença, o padrão de resposta cronotrópica em pacientes tendeu a ser próximo ao demonstrado por indivíduos normais. Esses dados demonstram depressão da capacidade de retração vagal durante o exercício em pacientes com hipertireoidismo, em associação com uma maior ativação do componente simpático em relação a indivíduos normais. Essas anormalidades funcionais parecem ser pelo menos parcialmente reversíveis quando os pacientes são controlados clinicamente com medicamentos antitireoidianos.^33^

Em suma, esses estudos demonstraram que a taquicardia em repouso observada em pacientes com hipertireoidismo tem uma importante contribuição da redução da atividade parassimpática eferente no nó sinusal, mas que essa contribuição não depende da participação significativa do componente simpático do sistema nervoso autônomo. Além disso, eles documentam a depressão da atividade parassimpática na taquicardia inicial do ED em pacientes com hipertireoidismo, bem como uma maior ativação simpática na fase tardia do exercício em comparação com indivíduos normais.

## Controle autonômico da frequência cardíaca em pacientes com prolapso da valva mitral

O controle autonômico da FC foi avaliado em pacientes com prolapso da válvula mitral em comparação a indivíduos normais, com os métodos de avaliação da magnitude da ASR em repouso e as respostas cronotrópicas ao ED. A arritmia sinusal foi de maior magnitude em pacientes com prolapso da válvula mitral quando comparados ao grupo controle; no entanto, as diferenças atingiram significância estatística apenas na frequência respiratória de 7 ciclos/min. Durante o ED na posição sentada (25, 50, 100, 150 W durante 4 min de forma gradual), a resposta da frequência cardíaca, seja em termos de taquicardia rápida precoce, dependente do vago (primeiros 10 s), ou taquicardia tardia, dependente do simpático (1-4 min), não foi significativamente diferente nos dois grupos estudados. Além disso, nenhuma diferença entre os grupos pôde ser detectada em relação à capacidade de exercício aeróbico, avaliada pela medição do limiar anaeróbico. Assim, nossos resultados mostram que neste grupo de pacientes do sexo masculino com prolapso da valva mitral e alguma redução da reserva cardíaca,^34^nenhuma anormalidade autonômica inquestionável de significado fisiológico pôde ser detectada, uma vez que o controle simpático e parassimpático da FC permanece normal durante o ED.^35^

## Regulação autonômica da frequência cardíaca após treinamento de exercícios de resistência

Indivíduos normais sedentários foram submetidos a 10 semanas de treinamento físico de resistência em cicloergômetro, após o qual apresentaram aumento do VO2máx e redução da FC em repouso na posição supina. O incremento da FC induzido pelo bloqueio farmacológico do sistema parassimpático com sulfato de atropina não diferiu significativamente antes e depois do treinamento de resistência. A magnitude da ASR também foi semelhante antes e depois do condicionamento físico obtido com o treinamento de resistência. Resultados semelhantes foram demonstrados pela avaliação do teste de ASR quando indivíduos sedentários foram comparados a corredores de média distância. Embora seja possível especular que a magnitude do valor da ASR não reflita necessariamente alterações no tônus parassimpático nas condições estudadas, os resultados atuais mostram claramente que valores aumentados de ASR não acompanham o aumento da capacidade de resistência. Além disso, a resposta da FC ao bloqueio farmacológico com atropina não sugeriu qualquer participação parassimpática na bradicardia em repouso induzida pelo treinamento físico.^36^Portanto, os mecanismos responsáveis pela bradicardia do treinamento de resistência em repouso em humanos permanecem controversos. Os dados disponíveis obtidos em cães com bloqueio farmacológico duplo (atropina + propranolol) e a variabilidade da FC foram consistentes com aumento da atividade parassimpática cardíaca, mas sem quaisquer alterações na frequência do nó sinusal intrínseco para explicar a bradicardia do treinamento em repouso.^37^

Em nosso laboratório, quando jovens sedentários foram submetidos a 10 semanas de treinamento físico de resistência, sentado em um cicloergômetro com freio eletromagnético, um aumento de 15% no consumo máximo de oxigênio (VO2máx) e uma redução de 16% na FC de repouso foram documentados. Antes e depois do treinamento, esses voluntários realizaram ED em um cicloergômetro em cargas de trabalho de 25, 50, 75, 100 e 150 W por 4 min em cada nível de exercício. Indivíduos saudáveis sedentários também foram comparados a atletas treinados (corredores de média distância). Durante os primeiros 10s de exercício, um período em que a taquicardia é mediada quase exclusivamente pela retirada vagal, os atletas apresentaram um aumento mais rápido na FC do que os indivíduos sedentários. A mesma tendência foi observada nos indivíduos sedentários após o período de treinamento, embora de menor magnitude. Durante a fase de ED em que a mediação simpática desempenha um papel importante (entre 30 s e 4 min), os atletas apresentaram menor aumento da FC do que os indivíduos sedentários, e o mesmo padrão de resposta foi observado no grupo sedentário que passou pelo treinamento físico de resistência. O aumento da FC em cada carga de trabalho de 0 a 4 minutos de ED foi menor em atletas do que em indivíduos sedentários e não foi alterado pelo treinamento de resistência em indivíduos sedentários. Esses resultados sugerem que o treinamento aeróbico diminui a contribuição simpática lenta tardia e aumenta a contribuição parassimpática rápida para a alteração da FC durante o ED, em comparação com a FC induzida pelas mesmas cargas de trabalho absolutas realizadas antes do treinamento aeróbico. Essas alterações funcionais no controle autonômico da FC podem ou não estar associadas a modificações nos valores absolutos da FC, que aumentam do repouso ao final do exercício. Em contraste com o que ocorre em atletas, as adaptações autonômicas observadas após o treinamento aeróbico de curta duração podem ocorrer durante o DE sem alterações na resposta geral da FC.^38^

Um padrão semelhante de adaptações fisiológicas após dez meses de treinamento aeróbico foi documentado em homens sedentários de meia-idade, que foi expresso principalmente como uma diminuição na influência simpática sobre a resposta da FC ao ED, que foi associada a um aumento na capacidade de transporte de oxigênio durante o esforço.^39^

Resumidamente, nossa investigação não indicou qualquer participação parassimpática na bradicardia em repouso induzida pelo treinamento físico de resistência. No entanto, durante o ED, atletas treinados apresentaram uma contribuição parassimpática aumentada para a alteração da FC em comparação com a FC induzida pelas mesmas cargas de trabalho absolutas realizadas anteriormente por indivíduos sedentários. Além disso, nossos resultados sugerem que o treinamento aeróbico diminui a contribuição simpática lenta tardia para a taquicardia.

## Função autonômica cardíaca em idosos saudáveis

A função autonômica foi avaliada em idosos saudáveis por meio do monitoramento da FC instantânea e das alterações da pressão arterial sistêmica em resposta à manobra de Valsalva, exercício isométrico, teste de pressão fria e administração de fenilefrina e nitrito de amila, induzindo aumento ou diminuição transitória aguda da pressão arterial sistêmica e bloqueio farmacológico com atropina. Apesar de uma ampla variedade de variáveis de confusão influenciarem as alterações do controle cardiovascular autonômico relacionadas à idade, nossos resultados sugerem a ocorrência de comprometimento parassimpático do controle da frequência cardíaca, embora os mecanismos globais envolvidos permaneçam desconhecidos.^40^

## Considerações finais

Os testes autonômicos padronizados descritos aqui, que utilizamos ao longo de várias décadas, são relativamente simples, reprodutíveis e confiáveis o suficiente para identificar disfunções na regulação autonômica da FC na ampla variedade de condições clínicas discutidas acima. Em geral, esses testes contribuíram para elucidar aspectos dos mecanismos fisiológicos ou fisiopatológicos subjacentes nesses cenários clínicos. No entanto, os distúrbios autonômicos detectados com esses testes não representam necessariamente o mecanismo mais importante da patogênese dessas doenças ou da condição fisiopatológica sob investigação. Por outro lado, tais testes podem ser úteis para excluir disfunções autonômicas cardiovasculares na maioria das condições fisiológicas, ou seja, para demonstrar que os indivíduos assim investigados são de fato normais, ou mesmo que seu comportamento ainda está dentro da normalidade na presença de estados patológicos. Como já mencionado acima, na doença cardíaca de Chagas, a implicação prognóstica do comprometimento autonômico não foi avaliada na ampla gama de investigações descritas. Entretanto, uma investigação recente do nosso laboratório relatou a identificação de um fator prognóstico significativo na doença cardíaca de Chagas crônica, relacionado à interrelação anormal entre os componentes de baixa e alta frequência na VFC.^25^

Além disso, embora a disfunção do sistema nervoso autônomo tenha sido bem documentada na fisiopatologia de diversas doenças cardiovasculares, a aplicabilidade clínica desses achados para fins terapêuticos é muito restrita. Muito provavelmente, essa limitação está relacionada, pelo menos em parte, ao fato de que a maioria dos estudos avaliou essencialmente o controle anormal da frequência cardíaca, enquanto é uma hipótese razoável que o significado prognóstico adverso da disautonomia possa ser mais dependente de distúrbios no nível ventricular. Assim, se descobriu que a inervação adrenérgica anormal no nível miocárdico se correlaciona com arritmia ventricular maligna na DC.^26^ Como este é de fato um achado intrigante, pesquisas adicionais são necessárias para elucidar o papel da disautonomia na maioria dos cenários clínicos investigados até o momento.

Também é digno de nota que os dados apresentados em relação a várias condições anormais devem refletir o conhecimento fisiopatológico prévio que existia quando os testes que avaliam o controle cardiovascular foram aplicados especificamente em nossos estudos. Portanto, se deve ter cautela ao traduzir esse conhecimento para os conceitos atuais. Por exemplo, pacientes com a chamada síndrome do prolapso da válvula mitral em nossos estudos estão agora incluídos na síndrome mais abrangente de disjunção do anel mitral.^41^ Esse conceito envolve o importante aspecto fisiopatológico referente à possibilidade de arritmia maligna em alguns dos pacientes,^42^ algo que não foi adequadamente abordado em estudos recentes, mas pode estar associado à ocorrência de disfunção autonômica.

Por fim, desde as primeiras investigações, tanto em modelos animais experimentais quanto em humanos, a disautonomia cardíaca tem sido associada à fisiopatologia da doença arterial coronariana, com profundas implicações para o prognóstico de pacientes que sofrem infarto do miocárdio.^43^ Além disso, a associação da variabilidade anormal da FC com a disfunção microvascular tem sido destacada em estudos recentes com pacientes diabéticos com insuficiência cardíaca, o que justificaria uma avaliação mais aprofundada para ser completamente compreendida.^44^
