# Supplementary material for: II – Avaliação do Controle Autonômico da Frequência Cardíaca em Diversas Condições Clínicas
Source: Arq Bras Cardiol. 2026 Jan 9;122(12):e20250112. [Article in Portuguese] doi: 10.36660/abc.20250112 (PMC12978365; doi:10.36660/abc.20250112)
Supplement: Translation

# Translation: II – Evaluation of Autonomic Control of Heart Rate in Various Clinical Conditions

**DOI:** 10.36660/abc.20250112i

**Authors:** Benedito Carlos Maciel, Lourenço Gallo-Jr, André Schmidt, José Antonio Marin-Neto

**Affiliations:** 1 Brazil Universidade de São Paulo – Campus Ribeirão Preto, Ribeirão Preto, SP – Brazil

**Keywords:** Autonomic Nervous System Diseases, Heart Rate, Sympathetic Nervous System, Parasympathetic Nervous System

## Abstract

Through the comparison of responses of normal volunteers, assessment of autonomic function under abnormal clinical conditions focused on the detection of dysautonomia involving both the parasympathetic and adrenergic limbs in patients with Chagas heart disease, post-cardiac surgery, chronic heart failure, mitral valve prolapse, and hyperthyroidism. In particular, the autonomic impairment observed in Chagas disease patients involved predominantly the parasympathetic control of heart rate at the sinus node level, and the adrenergic innervation at the myocardial ventricular level. The autonomic derangements observed in Chagas cardiomyopathy patients have only recently been explored in terms of their prognostic relevance, and their potential clinical implications for therapeutic purposes remain to be investigated.

Over the last nearly seven decades, our laboratory has accumulated significant expertise using several tests described above to evaluate autonomic control of heart rate, now focusing on various pathophysiological clinical conditions. The effect of endurance physical training and of aging was mostly focused on studies of normal volunteers whose responses at baseline served as controls to be compared during tests employed in individuals with some morbid clinical conditions. By far, the pathophysiology of Chagas heart disease involving the autonomic nervous system was the most predominant subject studied in our laboratory, since the early studies in the 1960s until the late studies just recently published in international journals (Central Illustration). Other pathological conditions focused on our studies were mitral valve prolapse, heart failure, post-cardiac surgery, and hyperthyroidism.

## Dysautonomia in Chagas’ disease

Consistent with anatomic parasympathetic denervation described by several independent investigators reporting on morphological studies, abnormal cardiac autonomic regulation has been conclusively demonstrated in Chagas’ disease (CD) patients.^1^Various pharmacological and physiological stimuli were used to show strikingly impaired parasympathetic heart rate (HR) regulation. The intravenous injection of hypertensive drugs such as metaraminol and phenylephrine was shown in patients with CD to produce comparable transient augmentation of SAP, without a similar degree of reflex bradycardia, in comparison to normal control individuals. CD patients also failed to respond with an HR increase to atropine intravenous administration. Dysautonomia evidenced with these pharmacological based tests was corroborated by other physiological tests that included facial water immersion, Valsalva maneuver, head-up and head-down tilt tests, respiratory sinus arrhythmia (RSA), handgrip, graded dynamic exercise, and spectral analysis of Holter recordings.^2 - 8^Overall, these studies showed that most CD patients are usually deprived of the tonic inhibitory action normally exerted by the parasympathetic system on the sinus node^9 , 10^( Figure 1 ). Moreover, these CD patients lack the vagally mediated mechanism to respond with rapid reflex bradycardia or tachycardia through parasympathetic withdrawal to transient changes in blood pressure or venous return. Furthermore, these studies paved the way for the concept of Chagas disease as a spontaneous experimental model adequate for studying a cardiomyopathy with a specific autonomic dysfunction at the sinus node level.^10^

**Figure 1:** – Effects of atropine sulfate on heart rate (peripheral venous administration at the dose of 0.04 mg/ kg). Effect of atropine on heart rate in normal controls and individuals with chronic Chagas cardiomyopathy. Absolute and mean values (left panel) and individual and mean incremental changes (right panel).12

It is important to emphasize that dysautonomia in CD is an early finding in the natural history of the disease, since, for example, abnormal HR responses to intravenous parasympathetic blockade with atropine have been consistently shown in CD preceding any signs of ventricular systolic dysfunction or heart failure. In contrast, the pharmacological sympathetic blockade with intravenous propranolol produced similar degrees of HR elevation in CD patients and normal controls.^9 , 10^

Baroreflex sensitivity was assessed using the method described by Pickering, Sleight et al.,^11^by relating the beat-to-beat pulse intervals to the immediately preceding systolic pressure values during transient changes in systemic arterial pressure induced by phenylephrine or amyl nitrite. In comparison to normal controls, both the bradycardia and the tachycardia observed in patients with CD were significantly blunted. However, the fast reflex bradycardia elicited by phenylephrine-induced hypertension was clearly more depressed than the fast reflex tachycardia hypotension caused by amyl nitrite. These findings were interpreted as indicating that the autonomic impairment in CD patients predominantly affected the parasympathetic limb – responsible for the bradycardia, whereas the sympathetic systemic – responsible for the tachycardia, occurring later, could be relatively spared^10^( Figure 2 ). These blunted chronotropic changes using pharmacological tests were corroborated by those observed in response to the Valsalva maneuver (40mm Hg for 20 seconds) in CD patients who showed no tachycardia in response to the intravenous atropine infusion. Despite having comparable pressure overshoot values after the Valsalva strain was released, CD patients who did not show HR increase after atropine also failed to have absolute bradycardia, and there was also a more delayed return of blood pressure to baseline values after the strain^1^( Figure 3 ).

**Figure 2:** – Baroreceptor sensitivity during acutely induced alterations in systemic vascular resistance by phenylephrine and amyl nitrite. Note the striking differences between normal and patients with cardiac Chagas disease during phenylephrine-induced hypertension; differences during amyl nitrite hypotension were less significant.10

**Figure 3:** – Arterial blood pressure (mean) and heart rate (mean and standard deviation) values during the Valsalva maneuver (40mm Hg for 20 seconds) in groups of normal individuals (A; n = 18) and patients with Chagas heart disease (B; n = 14). In comparison with normal controls, Chagas disease patients exhibit blunted heart rate changes during the strain and the blood pressure overshoot after the Valsalva maneuver. In the group of chagasic patients, there is no absolute bradycardia, and there is also a more delayed return of blood pressure to baseline values after the strain. The Chagas patients included in this study were selected because of no chronotropic response to atropine (0.04 mg/kg−1 intravenously).1

The reflex tachycardia induced by a change from the supine position to a 70^o^head-up tilt was studied in conscious normal individuals and patients with chronic Chagas’ heart disease in the absence of cardiac failure. CD patients showed markedly decreased HR responses during the initial 10 s following tilt to upright posture. A similar response was obtained in normals after parasympathetic blockade with atropine. Beta-adrenergic blockade failed to produce a significant effect on the initial HR response of normals, but the HR increment, at 1 and 5 min of tilt, was significantly reduced in normals and abolished in patients. These results in CD patients corroborate what had been found in normal individuals, of a biphasic mode of tachycardia elicited by the upright posture: initially it depends on parasympathetic withdrawal – a mechanism mostly depressed in the patients – while sympathetic stimulation becomes the predominant late mechanism for tachycardia In the orthostatic position.^12^( Figure 4 ) In these studies it also was seen that although less marked, CD patients additionally have some depression of the adrenergically mediated response to upright tilting.

**Figure 4:** – Effects of tilting normal subjects (green bars) and Chagas’ patients (blue bars) to the head-up position, before and after atropine and propranolol administration. In each instance, the mean value is indicated by the horizontal bar, and the standard error of the mean by the horizontal line at the top of the bar. Delta HR = increment of heart rate, 10s and 5 min after postural changes.12

However, not all patients with CD exhibit the abnormal parasympathetic control of HR described in the studies above. That this concept is true was shown in other investigations from our laboratory, when hemodynamic responses to sustained isometric exercise (handgrip at 30% of maximum voluntary capacity) were studied in different groups of patients with Chagas’s heart disease, all without previous or current heart failure. One group (1) of patients had profound impairment of parasympathetic control of HR, considering that they had no tachycardia in response to intravenous administration of atropine and no bradycardia during phase IV of the Valsalva maneuver. The other group (2) showed normal vagal regulation of HR, as judged by chronotropic responses to these tests. The HR change elicited by the handgrip test was significantly lower in group 1 than in group 2. Pressor responses and stroke index to handgrip were of similar magnitude in both groups. Cardiac output indexed for the body surface area increased during handgrip in group 2, but there was no significant change in group 1. Changes in measured systemic vascular resistance were significantly higher in group 1 than in group 2. This study also showed that parasympathetic impairment is associated with more conspicuous and adverse effects when there is inotropic depression of the myocardium, as shown by hemodynamic overload caused by isometric exercise in patients with Chagas’s heart disease. In such conditions, the blood pressure response to handgrip is predominantly mediated by an increase in systemic vascular resistance rather than by an increase in cardiac output^13^( Figure 5 ).

**Figure 5:** – Changes in heart rate (HR), mean systemic arterial pressure (SAP), cardiac index (ACI), and systemic vascular resistance (SVR) evoked by handgrip at 30% of maximal voluntary capacity in Chagas patients with (circles) and without parasympathetic impairment (triangles). Statistical significance was assessed by Student’s t-test for unpaired samples.13

Dynamic exercise was used with an electromagnetic braked cycle ergometer, to assess HR responses in relation to the other cardio-respiratory variables (pulmonary ventilation (V), oxygen consumption (VO2), carbon dioxide production (VCO2), and respiratory quotient (RQ)) comparing normal subjects with two groups of patients with chronic CD (with and without cardiomyopathy, i.e. with the indeterminate form of the disease). The time course of HR responses to the increasing workload was used to assess the functional conditions of the sympathetic and parasympathetic cardiac efferent limbs. Analyses of the results showed that (a) the group of CD patients with overt cardiomyopathy (abnormal electrocardiogram, even with normal cardiac size on chest X-rays, and no previous heart failure), had lower HRs increments (p < 0.05) than normal subjects during the initial 10 s of each exercise workload (delta HR 0-10 s) of effort (fast component of HR increase ); (b) differences between the normal control subjects and CD patients without cardiomyopathy were not statistically significant; (c) the abnormalities in the chronotropic responses are consistent with impairment of parasympathetic efferent modulation on the sinus node; (d) in contrast, the slow HR response (delta HR 1-4 min), which expresses the degree of sympathetic stimulation upon the sinus node, was comparable in the three groups studied, thus showing unimpaired adrenergic responses during dynamic exercise in the group of patients with the indeterminate form of CD; and (e) VO2, VCO2, and RQ values were normal at all workloads in both groups of individuals with CD, suggesting that vagal dysfunction does not affect oxygen transport at these submaximal levels of dynamic exercise^14^( Figure 6 ).

**Figure 6:** – Changes in heart rate induced by dynamic exercise at a) 0-10s and b) 1-4 min at all workloads (25, 50, 100, and 150W) in normal subjects and cardiac and non-cardiac Chagas disease patients. Solid lines denote medians, dashed lines the first and third quartiles, and vertical lines the most extreme data.14 CCC: cardiac Chagas disease; CNC: non-cardiac Chagas disease.

We also used power spectral analysis of heart rate variability (HRV), a most reliable non-invasive technique for assessing autonomic influence upon the sinus node in patients with CD ( Figure 7 ). A significant reduction of the potency of night and morning chronotropic variability, affecting both the low-frequency and high-frequency components, was demonstrated by spectral analysis of electrocardiographic 24-hour Holter recordings in CD patients. Thus, the attenuation of both components during spectral analysis of chronotropic variability in CD patients is consistent with the occurrence of both sympathetic and parasympathetic dysfunction. Moreover, this abnormal HRV has been documented in CD patients at rest, standing, or undertaking handgrip exercise.^8^

**Figure 7:** – Twenty-four-hour spectral variability of cardiac rate recorded in control subjects (N R) and in chronic Chagas disease patients (NA). Mean and standard deviation for the two groups. Green and block bars indicate sleeping (nighttime) and awakened (daytime) values, respectively. Clockwise from upper left: TP (total power), LFP (low frequency power), HFP (high frequency power), and LF/HF (ratio low/high frequency). Chagas patients showed lower values than normals, in addition to the lack of the expected LF/HF ratio, and awakening.28

As already pointed out above, the autonomic dysfunction in CD patients can be detected before the development of ventricular dysfunction and in all phases of the disease, even in those individuals with the indeterminate and isolated digestive forms^7 , 15 , 16^However, in some of our studies, there was a clear trend of patients showing parasympathetic cardiovascular dysfunction to have the typical CD digestive organ involvement, i.e., megaesophagus and or megacolon, whose pathophysiology is considered to be mostly due to visceral parasympathetic intramural denervation.^17 - 21^

Although the neurogenic theory had been proposed as the fundamental pathogenetic mechanism for Chagas’ cardiomyopathy,^22^the pathogenesis of Chagas cardiomyopathy is definitely more complex, and the neurogenic theory for Chagas cardiomyopathy met several unsolvable conceptual obstacles. In addition, detection of cardiac dysautonomia in all those studies was based essentially on assessing impaired HR responses, that was clearly caused by damage in the neuronal supply to the sinoatrial structures. Hence, adrenergic derangements occurring at the ventricular myocardial level may have been overlooked. A more detailed study from our laboratory focused on^123^iodine- metaiodobenzylguanidine (^123^I-MIBG) scintigraphy for evaluation of myocardial sympathetic nerve innervation and showed segmental sympathetic denervation that was detected even in patients with the indeterminate form of Chagas heart disease preceding left ventricular wall motion abnormalities^23^( Figure 8 ). An additional intriguing finding from that study was the increased^123^I-MIBG washout rate observed in patients with normal ventricular function.^24^This could be the result of paradoxically increased cardiac sympathetic activity at the early stages of Chagas disease.

**Figure 8:** – Examples of myocardial scintigraphy studies using I-123-MIBG (left column) and stress and/or redistribution thallium-201 (center and right columns, respectively). Polar map representation of percent myocardial uptake (related to maximum pixel count density) (upper row) and the result of quantitative analysis depicting areas of reduced uptake (>2.5 SDs in comparison to mean values of the control group) (lower row). (A) Thirty-seven-year-old man, group I Chagas’ disease patient (LV ejection fraction 50%). Reduced I-123-MIBG uptake in anteroseptal and apical septal areas is topographically related to reversible myocardial perfusion disturbance. (B) Fifty-four-year-old woman, group II Chagas’ disease (LV ejection fraction 52%). Large area of reduced I-123-MIBG uptake in the inferior, posterior, and apical walls that are topographically related to moderate fixed and/or paradox perfusion defect on the thallium-201 study. Severe wall motion impairment in the involved segments was also present.23

It is noteworthy that circulatory dysautonomia found in many patients with Chagas disease differs from other syndromes that affect the autonomic nervous system (e.g., diabetes mellitus, nervous Andrade amyloidosis), since no significant abnormality of the adrenergic vascular control that leads to symptoms or objective evidence of postural hypotension has been reported in those studies. Moreover, there has been no clear demonstration that autonomic impairment per se is prognostically relevant, and no therapeutic implications have been devised. In fact, although autonomic impairment has long been hypothesized as a potential mechanism causing sudden death in CD patients,^1^a link between reduced HRV and a more ominous prognosis associated with a high Rassi score,^25^has only recently been reported. Also, recent investigations from our laboratory provided evidence that the extent of patchy myocardial adrenergic denervation detected with^123^Iodine-MIBG is correlated with the occurrence and severity of malignant ventricular arrhythmia in patients with chronic Chagas cardiomyopathy.^26^

Of note, the degree of autonomic impairment is quite variable from individual to individual in the setting of CD, and in many studies, its magnitude was very subtle; thus, minuscule differences in comparison to normal controls may have been overinterpreted. Comparing the autonomic dysfunction of the sinus node detected by many different tests, with the sympathetic dysfunction of the left ventricle evaluated by^123^I-MIBG scintigraphy, in different groups of CD studied in our Lab, it seems that each one is of an independent nature and not interrelated. Overall, independent investigations point to 4 main pathogenetic mechanisms to explain the development of chronic Chagas cardiomyopathy, one of which is undoubtedly dysautonomia, and the other including microvascular disturbances, parasite-dependent myocardial aggression, and immune-mediated myocardial injury. Despite its prominent peculiarities, the role of autonomic derangements and microcirculatory disturbances is probably ancillary among the main causes of chronic myocardial damage. The pathogenesis of chronic Chagas heart disease is, in essence, dependent on a low-grade but incessant systemic infection with documented immune-adverse reaction. Hence, parasite persistence and immunological mechanisms are inextricably and causally related to myocardial aggression seen in the chronic phase of Chagas heart disease.^1^

In essence, many investigations employing various tests assessing the autonomic control of HR, with pharmacological and physiological methods, have shown marked disturbances of the parasympathetic system, and, to a lesser extent, also of the adrenergic system in patients with all forms of chronic Chagas disease. In addition, at the ventricular level, derangements in the adrenergic system could be detected, and, more relevant, these alterations may bear possible roles in triggering or aggravating cardiac arrhythmia.

## Autonomic regulation of heart rate in the syndrome of chronic heart failure due to various etiologies

Since the decade of 1970 two independent investigations reported abnormal autonomically mediated parasympathetic and sympathetic control of the HR in patients with ventricular dysfunction or congestive heart failure (CHF).^27 , 28^However, in contrast to what had been found in our studies with patients whose dysautonomia was due to CD and occurred in the absence of cardiac contractile dysfunction, these investigations on CHF could not report on any pathological evidence of abnormalities in the cardiac anatomic innervation. Hence, the impairment of baroreflex control of HR described in patients with CHF of whatever etiology could be functionally determined, nonspecific, and also possibly reversible. To determine if this derangement in this clinical setting of CHF could be reversible, our laboratory set out to assess the cardiac chronotropic control in patients with class IV chronic CHF of various etiologies before and after clinical compensation achieved by bed rest, salt restriction, diuretics, and vasodilators. The management was modified 3 days before the second autonomic evaluation, so as to reestablish the same diet and pharmacologic conditions of the previous baseline study. Documented clinical compensation led to a significant reduction in symptom-based NYHA class, body weight, and pulmonary and systemic congestion. Mean HR changes responses (beats/min) significantly increased after compensation in all patients tested with atropine, handgrip (30% maximum capacity), and head up tilting (5 minutes). A markedly attenuated baroreflex slope was confirmed to occur in the decompensated state of CHF, according to both phenylephrine and amyl nitrite tests. A striking increase in baroreflex sensitivity occurred after compensation, in both phenylephrine and amyl nitrite tests. These findings documented, for the first time in the literature, a reversible component of impaired baroreflex control of HR in severe CHF of nonspecific etiology, possibly due to its pronounced congestive effects.^29^

In summary, this investigation demonstrated that after clinical compensation of heart failure, there was a significant improvement in the autonomic control of HR documented by tests with atropine, handgrip, and head-up tilting, as well as by a remarkable increase in baroreflex sensitivity.

## Autonomic control of heart rate in patients treated with cardiac surgery under extracorporeal circulation

The sensitivity of the baroreceptor reflex to transient hypertension was determined in patients before (control) and after (72 hours) open cardiac operations with extracorporeal circulation (ECC). In all patients, early post-operative assessment of baroreflex sensitivity showed values that were appreciably decreased (p < 0.01) as compared to the preoperative sensitivity values ( Figure 9 ). The blunted sensitivity suggested severe impairment of baroreflex control of the sinoatrial node in the post-operative condition. The impaired baroreflex control did not correlate with concurrent alterations in HR or systemic arterial, left atrial, or right atrial pressures. In addition, respiratory sinus node arrhythmia was absent in all subjects. In four patients, subsequent studies at 4, 8, 10, and 12 months, respectively, after the open-heart operation, revealed good recovery of baroreflex sensitivity and respiratory influences on beat-to-beat variation. These findings point to further impairment of the fine control of HR imposed by the conditions of cardiac operations with ECC in patients undergoing this modality of thoracic surgery.^30^

**Figure 9:** – Individual baroreflex sensitivity values obtained with phenylephrine and amyl nitrite during decompensation (DECOMP.) and compensation (CGMP.) of congestive heart failure.29

Summarizing, this study showed that 72 hours after cardiac surgery with ECC, patients presented a marked impairment of the baroreflex control of the sinoatrial node and abolition of RSA, but presented a good recovery of these reflex controls of HR between 4 and 12 months after surgery.

## Heart rate autonomic regulation in patients with hyperthyroidism

The mechanisms that control resting HR in hyperthyroidism were evaluated in patients before and after treatment with propylthiouracil. Resting patients were studied under baseline conditions and following autonomic pharmacological blockade in two experimental sessions: first session, using propranolol (0.2 mg/kg body weight); second session, using atropine (0.04 mg/kg body weight) followed by propranolol (0.2 mg/kg body weight). All pharmacological blocking drugs were administered intravenously. Resting HR at baseline was significantly reduced after clinical and laboratory control of the disease. Following double blockade, the intrinsic HR was decreased after treatment (p < 0.025). The reduction in HR caused by propranolol was not significantly different before and after clinical control of hyperthyroidism with propylthiouracil. In contrast, atropine induced a higher elevation of HR after treatment than before clinical treatment. The present results suggest no appreciable participation of the sympathetic component of the autonomic nervous system in the tachycardia of hyperthyroidism, at least under the conditions of the present study. The small change observed in intrinsic HR, although significant, seems to indicate that this is not the most important mechanism involved in the tachycardia exhibited by patients with hyperthyroidism. Moreover, our results suggest that an important reduction in the efferent activity of the parasympathetic component participates in the mechanisms that modulate resting HR in hyperthyroidism.^31^This conclusion was confirmed when we evaluated the magnitude of the RSA in patients with hyperthyroidism, before and after treatment, showing that heart changes induced by this physiological maneuver significantly increased in all patients, thus indicating an important and reversible impairment of the efferent vagal activity upon the sinus node in human hyperthyroidism.^32^

We also investigated the relative contribution of the sympathetic and parasympathetic components of the autonomic nervous system to the regulation of the chronotropic response to dynamic exercise in seated position (for 4 min on electromagnetic braked cycle ergometer at levels of 5, 15, 25, 50, 75 W) that was evaluated indirectly in patients with thyrotoxicosis, before and after clinical control of the disease, and compared to normal volunteers, as well as under sympathetic pharmacological blockade with propranolol (0.2 mg/kg body weight).^33^

The magnitude of the net increase in HR evoked by each level of dynamic exercise was equivalent in normal and hyperthyroid patients, but in these patients a blunted increment occurred at the beginning of the exercise, which depends on a predominantly vagal withdrawal mechanism; in contrast, for each workload, after 30 s of effort, when sympathetic contribution becomes more the predominant mechanism for HR elevation, HR increment was larger in hyperthyroid patients compared to normal subjects.

In addition, we documented that in the hyperthyroidism patients, β-adrenergic blockage depressed tachycardia after 30 s of effort at the 15 and 50 W levels, whereas in normal individuals this effect was only manifested at 50 and 75 W. Furthermore, after clinical compensation of the disease, the pattern of chronotropic response in patients tended to be close to that shown by normal subjects. These data demonstrate depression of the capacity for vagal withdrawal during exercise in hyperthyroid patients, in association with a greater activation of the sympathetic component in relation to normal subjects. These functional abnormalities seem to be at least partially reversible when the patients are controlled clinically with anti-thyroid drugs.^33^

In short, these studies showed that resting tachycardia observed in patients with hyperthyroidism has an important contribution of reduced efferent parasympathetic activity on the sinus node, but that it is not dependent on significant participation of the sympathetic component of the autonomous nerve system. Additionally, they document depression of parasympathetic activity in the initial tachycardia of the dynamic exercise in hyperthyroid patients, as well as a greater sympathetic activation in the later phase of the exercise compared to normal individuals.

## Heart rate autonomic control in patients with mitral valve prolapse

The autonomic control of HR was evaluated in patients with mitral valve prolapse compared to normal subjects, with the methods of assessing the magnitude of RSA at rest and the chronotropic responses to dynamic exercise. Sinus arrhythmia was of higher magnitude in patients with mitral valve prolapse when compared to the control group; however, the differences reached statistical significance only at a respiratory frequency of 7 cycles/min. During dynamic exercise in seated position (25, 50, 100, 150 W during 4 min in a stepwise manner), the HR response, either in terms of the early, vagus-dependent, fast tachycardia (first 10 s), or the late, sympathetic-dependent tachycardia (1-4 min) was not significantly different in the two groups studied. Also, no differences between the groups could be detected in regard to aerobic exercise capacity, as evaluated by measurement of the anaerobic threshold. Thus, our results show that in this group of male patients with mitral valve prolapse and some curtailment of cardiac reserve,^34^no unquestionable autonomic abnormality of physiological significance could be detected, since the sympathetic and parasympathetic control of HR remains normal during dynamic exercise.^35^

## Heart rate autonomic regulation after endurance exercise training

Sedentary normal subjects were submitted to 10 weeks of endurance physical training on a cycle-ergometer, after which they showed an increase in V_O2_max and a reduction in resting HR when resting in the supine position. The HR increment elicited by pharmacological blockade of the parasympathetic system with atropine sulphate was not significantly different before and after the endurance training. The magnitude of RSA was also similar before and after the physical conditioning attained with the endurance training. Similar results were shown by assessing the RSA test when sedentary individuals were compared with medium-distance athletic runners. Although it is possible to speculate that the magnitude of RSA value does not necessarily reflect changes in parasympathetic tonus under the conditions studied, the present results clearly show that augmented RSA values do not accompany increased endurance capacity. In addition, the HR response to pharmacological blockade with atropine did not suggest any parasympathetic participation in the resting bradycardia induced by physical training.^36^Therefore, the mechanisms responsible for the resting endurance training bradycardia in humans remain controversial. Available data obtained in dogs using double pharmacological blockade (atropine + propranolol) and HR variability were consistent with enhanced cardiac parasympathetic activity but without any changes in the intrinsic sinus node rate to explain resting training bradycardia.^37^

In our lab, when young sedentary subjects were submitted to 10 weeks of endurance physical training, seated on an electromagnetic braked cycle ergometer, a 15% increase in maximal oxygen consumption (VO2max) and a 16% reduction in resting HR were documented. Before and after training, these volunteers performed dynamic exercise (DE) on a cycle-ergometer at workloads of 25, 50, 75, 100, and 150 W for 4 min at each level of exercise. Sedentary healthy subjects were also compared to trained athletes (medium-distance runners). During the first 10 seconds of exercise, a period when tachycardia is mediated almost exclusively by vagal withdrawal, the athletes presented a more rapid increase in HR than sedentary subjects. The same tendency was observed in the sedentary individuals after the training period, although of a lesser magnitude. During the dynamic exercise phase in which sympathetic mediation plays an important role (between 30s and 4 min), the athletes presented lower HR increase than the sedentary individuals, and the same response pattern was observed in the sedentary group who underwent the endurance physical training. The HR increase at each workload from 0 to 4 min of dynamic exercise was lower in athletes than in sedentary subjects and was not changed by endurance training in sedentary subjects. These results suggest that aerobic training decreases the late slow sympathetic and increases the fast parasympathetic contribution to HR change during dynamic exercise in comparison with the HR elicited by the same absolute workloads performed before the aerobic training. These functional changes in the autonomic control of HR may or may not be associated with modifications of absolute HR values, which increase from rest to the end of exercise. In contrast to what happens in athletes, the autonomic adaptations observed after short-term aerobic training may occur during DE without changes in the overall HR response.^38^

A similar pattern of physiological adaptations after ten months of aerobic training was documented in sedentary middle-aged men, which was mainly expressed as a decrease in the sympathetic influence upon HR response to dynamic exercise, which was associated with an increase in oxygen transport capacity during effort.^39^

Concisely, our investigation did not indicate any parasympathetic participation in the resting bradycardia induced by endurance physical training. However, during dynamic exercise, trained athletes showed an increased parasympathetic contribution to HR change in comparison with the HR elicited by the same absolute workloads performed before by sedentary subjects. In addition, our results suggest that aerobic training decreases the late slow sympathetic contribution to tachycardia.

## Cardiac autonomic function in healthy elderly people

Autonomic function was evaluated in healthy elderly subjects by monitoring instantaneous HR and systemic arterial pressure changes in response to Valsalva Maneuver, isometric exercise, cold pressure test, phenylephrine and amyl nitrite administration, inducing acute transient increase or decrease in systemic arterial pressure, and pharmacological blockade with atropine. Despite the fact that a wide variety of confounding variables influences age-related changes of autonomic cardiovascular controlour results suggest the occurrence of parasympathetic impairment of HR control, although the global mechanisms involved remain unknown.^40^

## Closing remarks

The standardized autonomic tests described here, which we have used over several decades, are relatively simple, reproducible, and reliable enough to identify dysfunction in the autonomic regulation of HR in the wide variety of clinical conditions discussed above. In general, these tests contributed to elucidating aspects of the underlying physiological or pathophysiological mechanisms in these clinical settings. However, the autonomic derangements detected with these tests do not necessarily represent the most important mechanism of the pathogenesis of these diseases or the pathophysiological condition under investigation. On the other hand, such tests can be useful to exclude cardiovascular autonomic dysfunction in most physiological conditions, that is, to demonstrate that the individuals thus investigated are indeed normal, or even that their behavior is still within normal range in the presence of disease states. As already mentioned above, in Chagas’ heart disease, the prognostic implication of autonomic impairment has not been assessed in the wide range of investigations described. However, one recent investigation from our laboratory has reported the identification of a significant prognostic factor in chronic Chagas’ heart disease as related to the abnormal interrelation between the low and high frequency components in the HRV.^25^

Moreover, even though the autonomic nervous system dysfunction has been well documented in the pathophysiology of several cardiovascular diseases, the clinical applicability of these findings for therapeutic purposes is very restricted. Most probably, this limitation is related, at least partially, to the fact that most studies evaluated essentially the abnormal control of HR, whereas it is a reasonable hypothesis that the adverse prognostic significance of dysautonomia may be more dependent on derangements at the ventricular level. Thus, abnormal adrenergic innervation at the myocardial level has been found to correlate with malignant ventricular arrhythmia in CD.^26^Because this is indeed an intriguing finding, further research is warranted to elucidate the role of dysautonomia in most clinical settings investigated thus far.

It is also noteworthy that the data presented in regard to various abnormal conditions must reflect the background pathophysiological knowledge that existed when the tests evaluating the cardiovascular control were applied, specifically in our studies. Thus, caution must be taken to translate that knowledge to current concepts. For example, patients with so-called mitral valve prolapse syndrome in our studies are now included in the most comprehensive syndrome of mitral annulus disjunction.^41^This concept entails the important pathophysiological aspect concerning the possibility of malignant arrhythmia in some of the patients,^42^something that has not been adequately addressed in recent studies but may be associated with the occurrence of autonomic dysfunction.

Finally, since early investigations both in experimental animal models and in humans, cardiac dysautonomia has been linked to the pathophysiology of coronary artery disease, with profound implications for the prognosis of patients suffering myocardial infarction.^43^Also, the association of abnormal HRV with microvascular dysfunction has been highlighted in recent studies of diabetic patients with heart failure. It would warrant further evaluation to be completely understood.^44^
